# Atypical lignification in eastern leatherwood (*Dirca palustris*)

**DOI:** 10.1111/nph.16394

**Published:** 2020-02-03

**Authors:** Yaseen Mottiar, Notburga Gierlinger, Dragica Jeremic, Emma R. Master, Shawn D. Mansfield

**Affiliations:** ^1^ Department of Wood Science University of British Columbia 2424 Main Mall Vancouver BC V6T 1Z4 Canada; ^2^ Department of Nanobiotechnology Institute for Biophysics University of Natural Resources and Life Sciences Vienna Muthgasse 11 Vienna 1190 Austria; ^3^ Department of Sustainable Bioproducts Mississippi State University Box 9680 Starkville MS 39759 USA; ^4^ Department of Chemical Engineering & Applied Chemistry University of Toronto 200 College Street Toronto ON M5S 3E5 Canada

**Keywords:** eastern leatherwood (*Dirca palustris*), lignin, middle lamella, model of lignification, secondary cell walls, wood flexibility

## Abstract

- Lignin is a complex phenolic biopolymer found mainly in the secondary cell walls of vascular plants, where it contributes to mechanical strength, water conduction, and plant defence. We studied the lignin of eastern leatherwood (*Dirca palustris*) because this slow‐growing woody shrub is known for its flexible stems.
- Various analytical techniques and microscopy methods were employed to examine the composition and distribution of lignin and structural polysaccharides in leatherwood xylem in comparison with trembling aspen (*Populus tremuloides*) and white spruce (*Picea glauca*).
- We found that leatherwood has low overall levels of lignin, a high syringyl lignin content, and a unique distribution of lignin. Most remarkably, the cell corners and middle lamellae remain unlignified in mature xylem.
- These findings help explain the flexibility of leatherwood and also call into question the classical model of lignification, which purports that lignin polymerization begins in the cell corners and middle lamellae. This atypical lignification regime vividly illustrates the diversity in plant secondary cell wall formation that abounds in nature and casts leatherwood as a new model for the study of lignin biogenesis.

## Introduction

Eastern leatherwood (*Dirca palustris* L.) is a slow‐growing woody angiosperm found sporadically across eastern North America in rich, mesic soils of hardwood and mixed forests (Zasada *et al.*, 2008). Leatherwood, a member of the family Thymelaeaceae, was traditionally used by indigenous peoples and early settlers for natural cordage as the stems are remarkably flexible (Thoreau, 1856; Supporting Information Fig. S1). Much of the contemporary work has focused on this species' horticultural potential and ecological context due to its attractive arboresque growth form and because the ephemeral flowers emerge in early spring when pollinator activity is limited (Williams, 2004; Peterson & Graves, 2011).

The earliest recorded description of leatherwood was in 1700 by the Canadian physician and naturalist Michel Sarrazin (Mottiar, 2012). Nearly 200 yr later, the French botanist Van Tiegham first reported weakly lignified cell walls in various other members of the family Thymelaeaceae (Van Tieghem, 1893). It has been suggested that poor lignification of the xylem is the cause of leatherwood's flexibility (Nevling, 1962), but this has not been thoroughly examined. As lignin is both biologically and industrially important, a careful study of plants with atypical lignification could help address outstanding questions in lignin biology and could potentially inspire ongoing efforts to breed or engineer commercially relevant species for key industrial applications.

Lignin is a phenolic polymer found mainly in the secondary walls of plant cells, where it contributes to structural integrity, xylem conductance, and plant defence (Weng & Chapple, 2010). Since lignin represents a significant investment of photosynthate‐derived carbon, its biosynthesis is tightly regulated in a spatio‐temporal manner. Accordingly, lignin content and composition vary widely between cell types, throughout plant development, among plant taxa, and across different environmental conditions (Boerjan *et al.*, 2003).

Lignin biosynthesis occurs in the plastid and cytosol of plant cells via the shikimate and phenylpropanoid pathways, which give rise to *p*‐coumaryl, coniferyl, and sinapyl alcohols, the primary lignin precursors. These three monolignols are subsequently exported to the apoplast, where enzyme‐catalysed dehydrogenation yields radical monomers that undergo coupling and cross‐coupling reactions to produce polymeric lignin with *p*‐hydroxyphenyl, guaiacyl, and syringyl units, respectively (Ralph *et al.*, 2004). Perturbation in the supply of monolignols leads to lignin polymers with altered composition and structure, and non‐canonical monomers can also be incorporated (Mottiar *et al.*, 2016).

The prevailing model of lignification recognizes three phases: lignin initiation in the cell corners and middle lamellae, slow lignification during secondary cell wall formation, and rapid lignification following the deposition of cellulose and hemicellulose (Wardrop, 1957; Donaldson, 2001). As the cell corners and middle lamellae are the first to become lignified, it has been hypothesized that polymerization begins at discrete nucleation sites located in these regions (Terashima, 1990; Donaldson, 1994). Although ferulate moieties bound to glucuronoarabinoxylan have been described as potential nucleation sites in the cell walls of grasses (Ralph *et al.*, 1995), no universal initiation mechanism has yet been identified.

Surveys of nature's diversity have helped demystify the molecular underpinnings of lignification, but much of the focus has been on model species, and reports of woody plants with unusual lignification remain sparse. Herein, we describe the cell wall composition of leatherwood and reveal a unique distribution of lignin throughout the xylem and across the cell wall. Together, these results portray a remarkable example of atypical lignification that diverges from the classical model of lignin deposition and cell wall organization.

## Materials and Methods

### Wood samples

Woody stems were harvested from mature plants of *D. palustris* L. (eastern leatherwood) near Pembroke, Ontario (45°49′N, 77°6′W). Similarly, stems of *Populus tremuloides* Michx. (trembling aspen) and *Picea glauca* (Moench) Voss (white spruce) were collected as reference samples. Upright‐growing stems were chosen to avoid any reaction wood, and all stems were devoid of any signs of damage or attack by pests or pathogens. Three biological replicates were harvested for each species from the same wood lot. All stems were between 1 and 3 cm in diameter, and samples used for microscopy and imaging were taken near the base. Additional samples of *D. palustris*, *Dirca occidentalis*, *Dirca decipiens*, and *Dirca mexicana* were generously provided by Z. Hudson (Iowa State University).

### Analysis of lignin and structural polysaccharides

Established protocols were followed for Fourier‐transform infrared (FTIR) spectroscopy (potassium bromide pellets), lignin content (Klason lignin), structural polysaccharides composition (ultra‐high‐performance liquid chromatography with diode array detection of benzocaine‐derivatized acid hydrolysates), acetyl content (high‐performance liquid chromatography with refractive index detection of alkaline hydrolysates), lignin composition (gas chromatography with flame ionization detection of thioacidolysis reaction products), cellulose content (alpha cellulose), cell wall crystallinity (reflection mode X‐ray diffraction), and cellulose microfibril angle (differential interference contrast microscopy). Detailed descriptions of the methodology are provided in Methods S1.

### Microscopy

Stem specimens for microscopy were sectioned using a sliding block microtome. A droplet of water was placed on the blade to maintain sample integrity while preparing 15 μm thick sections. Bright‐field microscopy was performed using a Zeiss Stemi 2000‐C stereomicroscope (Carl Zeiss AG, Oberkochen, Germany) and a Leica DMR compound microscope (Leica Microsystems GmbH, Wetzlar, Germany) equipped with a Canon EOS Rebel T5 digital camera (Canon Inc., Tokyo, Japan). Confocal microscopy was performed to visualize lignin autofluorescence in unstained sections using an Olympus FV1000 MPE laser scanning microscope (Olympus Corp., Tokyo, Japan) equipped with a 405 nm excitation laser and emission filter sets spanning 425–525 nm. Images were cropped and scale bars were added using Adobe illustrator (Adobe Systems Inc., San Jose, CA, USA), and pixel intensity plots were generated using ImageJ (National Institutes of Health, Bethesda, MD, USA).

Double histochemical staining of lignified and nonlignified cell walls was achieved using safranin‐O (1% dye in water) and astra blue (1% dye in 2% tartaric acid). After a 2 min incubation, sections were washed thoroughly with distilled water and examined by light microscopy. Staining of syringyl units in lignin was performed using the Mäule reaction. After a 5 min incubation in 0.5% potassium permanganate, sections were washed thoroughly with distilled water and incubated in 3.7% hydrochloric acid for an additional 2 min. Sections were then transferred to concentrated ammonium hydroxide and examined by light microscopy. Staining of lignin‐associated aldehyde units was achieved using the Wiesner reaction with phloroglucinol–hydrochloric acid. After a 2 min incubation in freshly prepared stain (2 : 1 mixture of 3% aqueous phloroglucinol dye with concentrated hydrochloric acid), sections were examined by light microscopy.

### Confocal Raman imaging

A CM3050 S cryomicrotome (Leica) was used to prepare 16 µm thick sections for imaging with an Alpha300RA Raman instrument (WITec GmbH, Ulm, Germany) as described previously (Gierlinger *et al.*, 2012). The samples were excited using a linear polarized VIS laser (*λ*_ex_ = 532 nm, laser power 30 mW) via a 100× oil immersion objective lens (Zeiss, numerical aperture 1.4, coverslip correction 0.17 mm). Raman signals were backscattered through the same objective, directed through a 50 µm optical multifibre to a UHTS 300 spectrometer (WITec) fitted with a 600 g mm^−1^ grating, and finally to a DU401 BV CCD camera (Andor Technology Ltd, Belfast, UK). control four acquisition software (WITec) was used for data collection with 0.3 µm steps at 0.07 s per spectrum, and the Project four plus software (WITec) was used for data analysis. After cosmic ray removal, Raman images were calculated based on band integration, and a hierarchical cluster analysis was performed as described previously (Gierlinger *et al.*, 2012).

### Time‐of‐flight secondary ion mass spectrometry imaging

Stem specimens for time‐of‐flight secondary ion mass spectrometry (ToF‐SIMS) analysis were extracted and solvent‐exchange‐dried with ethanol sequentially from 30% to 95% and then sectioned using a rotary microtome equipped with a diamond knife. Spectra were recorded by Surface Interface Ontario (University of Toronto) using a ToF‐SIMS IV instrument (ION‐TOF GmbH, Münster, Germany) employing the scan parameters reported previously (Goacher *et al.*, 2011). Since lignin contributes considerable charge background, low‐energy electron flooding was used to facilitate the collection of positively charged secondary ions. Nominal mass resolution spectra were obtained using a pulsed current of *c.* 0.1 pA. Spectra were calibrated to the C^+^, CH_3_^+^, C_2_H_3_^+^, C_2_H_5_^+^ and C_7_H_7_^+^ ions using the surface lab 6.1 software (ION‐TOF). False‐colour ion images were then assembled with lignin ions being those reported previously as ‘Group 1’ assignments by Goacher *et al.* (2011) as well as *m*/*z* 167 and 181 for syringyl lignin and 107 and 121 for *p*‐hydroxyphenyl lignin moieties, and structural polysaccharides being those reported as ‘Group 2’ assignments with the addition of *m*/*z* 115 and 133 for xylan.

## Results

### Fourier transform infrared spectroscopy

**Figure 1:**
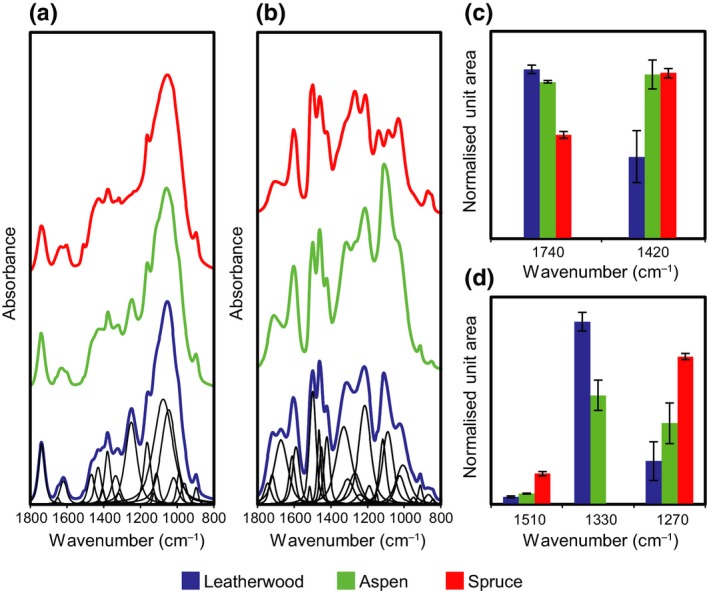
Semi‐quantitative Fourier‐transform infrared spectroscopy. Peak fitting of spectra from (a) chlorite holocellulose and (b) acid‐insoluble lignin derived from leatherwood (blue), aspen (green), and spruce (red) xylem with deconvoluted peaks shown only for leatherwood (black). Responses for several wavenumbers are plotted by normalized unit area for (c) chlorite holocellulose and (d) acid‐insoluble lignin. Three replicates were analysed for each species and sample type, and the error bars show standard deviation. No fitted peak was obtained for spruce at 1330 cm^−1^.

We initially used FTIR spectroscopy to survey the chemical composition of leatherwood xylem in comparison to samples of aspen (*P. tremuloides*) and spruce (*P. glauca*). These taxa were chosen for comparison because their cell wall composition and xylem histology have been studied extensively (Pettersen, 1984; Schweingruber *et al.*, 2011). Principal component analysis was used to identify chemical features unique to leatherwood (Fig. S2; Notes S1). To further investigate key spectral differences between the three species, curve fitting and a semi‐quantitative analysis were then performed using spectra recorded on preparations of chlorite holocellulose and acid‐insoluble lignin (Fig. 1a,b).

Leatherwood holocellulose produced a stronger signal at 1740 cm^−1^ corresponding to the C=O stretching mode of esters and unconjugated ketones compared to aspen and spruce, pointing to higher levels of wall‐bound acetate or uronic acids in leatherwood (Fig. 1c; see Table S1 for annotations of FTIR wavenumbers). In addition, leatherwood gave a considerably weaker response at the 1420 cm^−1^ wavenumber, which has been ascribed to the CH_2_ bending mode of cellulose, suggesting that leatherwood may have lower cell wall crystallinity or less cellulose.

Curve fitting of the lignin spectra revealed weaker signals for leatherwood at 1510 and 1270 cm^−1^ compared with spruce, and a stronger response at 1330 cm^−1^ compared with aspen (Fig. 1d). The 1510 cm^−1^ wavenumber corresponds to aromatic skeletal vibrations in lignin, whereas the 1270 and 1330 cm^−1^ bands originate from the ring‐breathing and C–O stretching modes of guaiacyl and syringyl lignin, respectively, suggesting that leatherwood xylem may have less lignin and a higher ratio of syringyl : guaiacyl lignin units.

### Lignin content and composition

To corroborate the FTIR results, we measured the Klason lignin content by two‐stage acid hydrolysis and analysed the lignin composition using thioacidolysis (Table 1). In this analysis, leatherwood had less than half as much acid‐insoluble lignin as spruce and 37% less than aspen. On the other hand, leatherwood produced more than 12 times as much acid‐soluble lignin as spruce and 33% more than aspen. In addition, *p*‐hydroxyphenyl units were absent in leatherwood, and the lignin was found to contain 80% syringyl units compared with 71% in aspen.

**Table 1:** Xylem cell wall composition and physicochemical properties.

Cell wall properties	Leatherwood	Aspen	Spruce
**Lignin content (% w/w)**			
Acid‐insoluble lignin	11.30 ± 0.19	17.96 ± 0.31	28.54 ± 1.30
Acid‐soluble lignin	5.24 ± 0.23	3.92 ± 0.06	0.43 ± 0.02
Total lignin content	16.61 ± 0.25	21.91 ± 0.30	28.96 ± 1.32
**Lignin composition (% w/w)**			
*p*‐Hydroxyphenyl	Not detected	0.53 ± 0.11	2.38 ± 0.35
Guaiacyl	19.53 ± 1.11	28.65 ± 0.38	97.62 ± 0.35
Syringyl	80.47 ± 1.11	70.82 ± 0.27	Not detected
**Structural polysaccharides (% w/w)**			
Glucose	35.00 ± 0.32	45.87 ± 0.36	42.84 ± 1.77
Xylose	23.19 ± 0.37	16.69 ± 0.42	7.19 ± 0.12
Mannose	0.66 ± 0.03	2.01 ± 0.08	10.15 ± 0.56
Galactose	1.14 ± 0.05	0.68 ± 0.04	2.37 ± 0.50
Arabinose	1.60 ± 0.10	0.52 ± 0.04	1.61 ± 0.15
Rhamnose	0.84 ± 0.04	0.49 ± 0.03	0.25 ± 0.05
Galacturonic acid	3.52 ± 0.13	1.37 ± 0.06	1.06 ± 0.19
Glucuronic acid	0.77 ± 0.11	0.38 ± 0.03	0.36 ± 0.07
4‐*O*‐Methyl glucuronic acid	2.59 ± 0.05	1.65 ± 0.04	1.36 ± 0.06
**Other cell wall properties**			
Cellulose content (% w/w)	31.44 ± 0.66	36.61 ± 2.42	35.49 ± 0.54
Ash content (% w/w)	1.20 ± 0.18	0.44 ± 0.07	0.19 ± 0.08
Wall‐bound acetyl (% w/w)	5.74 ± 0.13	4.58 ± 0.04	1.29 ± 0.05
Crystallinity index	0.31 ± 0.01	0.34 ± 0.02	0.37 ± 0.02
Microfibril angle (°)	57.9 ± 1.9	32.1 ± 5.4	39.5 ± 1.9

Three biological replicates were analysed in triplicate, and standard deviations are shown alongside mean values.

### Structural polysaccharides

On account of the comparatively lower lignin content of leatherwood xylem, we considered whether the structural polysaccharides might also be altered (Table 1). This analysis revealed that leatherwood has more xylose (39% more than aspen, 322% more than spruce) and contains more rhamnose (70% more than aspen, 329% more than spruce), but has less glucose (24% less than aspen, 18% less than spruce) and mannose (67% less than aspen, 93% less than spruce). Leatherwood also has more galactose and arabinose than aspen (68% and 305%, respectively) and less galactose than spruce (52%). Furthermore, alkaline hydrolysis of cell‐wall‐bound acetate revealed that leatherwood xylem contains 25% more acetyl groups than aspen and four times more than spruce.

Leatherwood has twice as much glucuronic acid as aspen and spruce, and about 1.5 times as much 4‐*O*‐methyl glucuronic acid (Table 1). In addition, leatherwood contains 2.6 times more galacturonic acid than aspen and 3.3 times more than spruce, pointing to higher levels of pectin in leatherwood xylem. It should be emphasized that these values are almost certainly underestimates, as uronic acids are not completely liberated during sulphuric acid hydrolysis (Willför *et al.*, 2009).

The alpha cellulose procedure was used to quantify the amount of cellulose, and X‐ray diffraction and differential interference contrast microscopy were used to evaluate the physical properties of cellulose microfibrils (Fig. S3; Table 1; Notes S1). The cellulose content of leatherwood was found to be 14% less than aspen and about 11% less than spruce, and the crystallinity index was 11% lower than aspen and 16% lower than spruce. On the other hand, leatherwood xylem showed substantially larger microfibril angles than the other species.

### Cell wall histochemistry

**Figure 2:**
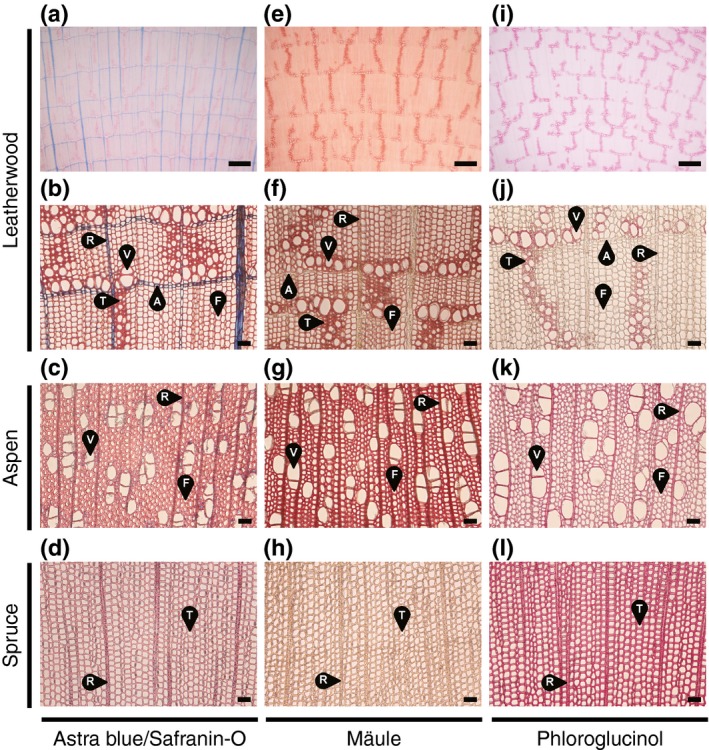
Lignin histochemical analysis. Transverse cross‐sections of leatherwood, aspen, and spruce xylem stained with (a–d) astra blue and safranin‐O, (e–h) by the Mäule reaction, and (i–l) with phloroglucinol–hydrochloric acid. Images show vessel elements (V), fibres (F), ray parenchyma (R), axial parenchyma (A), and tracheids (T). Two magnifications are shown for leatherwood. Bars: (a, e, i) 200 μm; (b–d, f–h, j–l) 10 μm.

We next set out to dissect the histology of leatherwood xylem in light of its notable cell wall composition. In addition to the unlignified ray parenchyma cells, astra blue staining showed concentric rings of unlignified axial parenchyma laid down at the end of each growth cycle (Fig. 2a,b). Safranin‐O uncovered lignin‐rich vessel elements in oblique strands amongst smaller thick‐walled tracheids forming an unusual dendritic pattern within a field of libriform fibres. For comparison, staining of aspen showed small clusters of vessels arranged diffusely amidst fibres and ray parenchyma (Fig. 2c), whereas tracheids and rays predominated in spruce xylem (Fig. 2d).

To ascertain whether lignin composition varies in a cell‐type‐specific manner in leatherwood, we examined cross‐sections using the syringyl lignin‐specific Mäule reaction. Axial and ray parenchyma remained unstained, whereas fibres, vessels, and vasicentric tracheids appeared to be stained equally (Fig. 2e,f). Staining of vessels in aspen was weaker than fibres, indicating less syringyl lignin, as expected (Fig. 2g), and spruce cell walls were not stained due to the absence of syringyl lignin in gymnosperms (Fig. 2h).

Staining with phloroglucinol was confined primarily to the strands of vessels and vasicentric tracheids in leatherwood (Fig. 2i,j), suggesting a difference in the composition of lignin‐associated aldehydes between the walls of fibres and those of vessels and vasicentric tracheids. No such patterns were observed in aspen or spruce, but the overall level of staining was more intense, reflecting the inherently higher lignin concentrations (Fig. 2k,l).

### Label‐free chemical imaging

**Figure 3:**
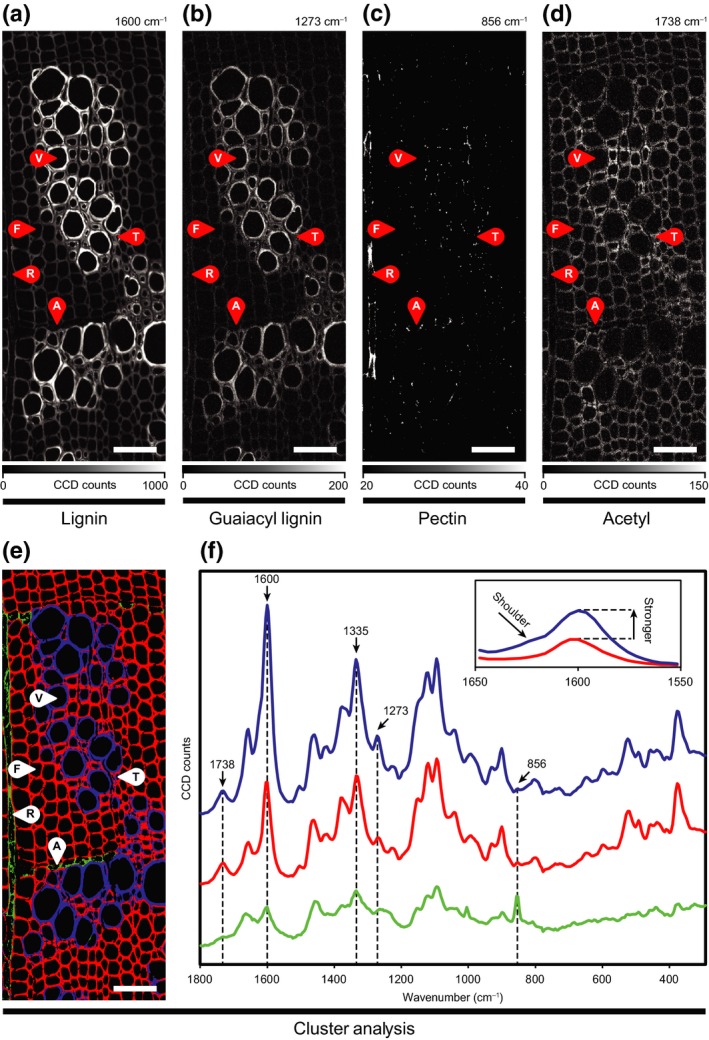
Label‐free chemical imaging. Transverse cross‐sections of leatherwood xylem imaged by confocal Raman microscopy. Chemical images are shown for specific integrated bands corresponding to (a) total lignin (1600 cm^−1^), (b) guaiacyl lignin (1273 cm^−1^), (c) pectin (856 cm^−1^), and (d) acetyl (1738 cm^−1^). (e) A false‐colour image and (f) corresponding Raman spectra are shown from the cluster analysis to highlight the spectral differences between vessels and vasicentric tracheids (blue), fibres (red), and parenchyma (green). The inset in (f) shows zoomed‐in spectra normalized at 2920 cm^−1^. Images show vessel elements (V), fibres (F), ray parenchyma (R), axial parenchyma (A), and tracheids (T). Bars, 50 μm.

To further resolve the cell‐type‐specific differences in cell wall composition, high spatial‐resolution Raman imaging was performed on leatherwood xylem cross‐sections. The signal at 1600 cm^−1^ corresponds to aromatic skeletal vibrations in lignin and can be used to examine the distribution of lignin (Gierlinger *et al.*, 2012). Integrating this band revealed stronger signals in vessels and vasicentric tracheids, pointing to a difference in lignin content and/or composition (Fig. 3a). An even more drastic difference was seen with the guaiacyl lignin‐sensitive band centred around 1273 cm^−1^ (Agarwal *et al.*, 2011), suggesting that vessels and vasicentric tracheids have more lignin and particularly more guaiacyl units (Fig. 3b). The signal at 856 cm^−1^, corresponding to C6–C5–O5–C1–O1 backbone vibrations of pectin (Synytsya *et al.*, 2003), was confined to cell corners and middle lamellae, and to the walls of ray and axial parenchyma (Fig. 3c). Finally, the band at 1738 cm^−1^, ascribed to C=O stretching of ester and acetyl groups (Agarwal *et al.*, 2011), was most prominent in the cell walls of vasicentric tracheids, but was also present in vessels and fibres (Fig. 3d).

In order to delve deeper into the spectral differences between cell types in leatherwood xylem, we performed a cluster analysis on the Raman hyperspectral dataset and identified three pixel clusters corresponding to the cell walls of vessels, fibres and vasicentric tracheids, as well as ray and axial parenchyma (Fig. 3e). Examination of the corresponding spectra (Fig. 3f) revealed that parenchyma differ broadly from vessels, tracheids and fibres having less of the acetyl (1735 cm^−1^) and lignin‐specific signals (1600, 1335, 1273 cm^−1^), but more of the pectin‐specific band (856 cm^−1^). This analysis also confirmed that vessels have more lignin than fibres and tracheids due to a much stronger response near 1600 cm^−1^. Comparing these spectra also showed that vessel cell walls have more guaiacyl lignin due to a greater response at 1273 cm^−1^ and, in accordance with the phloroglucinol staining results, more aldehyde groups revealed by the shoulder at 1620 cm^−1^, which corresponds to the C=C stretching mode of aldehydes (Fig. 3f inset; Agarwal *et al.*, 2011).

### Lignin‐deficient middle lamellae

**Figure 4:**
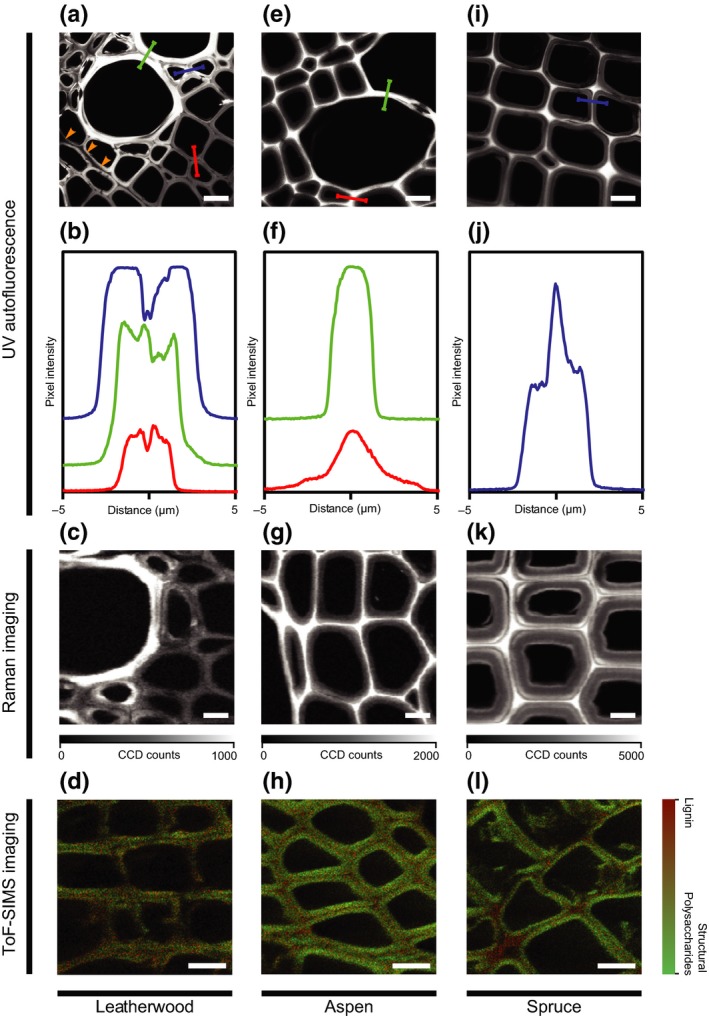
Lignin distribution across adjacent cell walls. Transverse cross‐sections of (a–d) leatherwood, (e–h) aspen, and (i–l) spruce xylem viewed by (a, e, i) ultraviolet (UV) microscopy, (c, g, k) confocal Raman imaging, and (d, h, l) time‐of‐flight secondary ion mass spectrometry (ToF‐SIMS). Pointwise signal intensity of lignin autofluorescence is shown (b, f, j) for line traces (a, e, i) across adjacent cell walls of fibres (red), vessels (green), and tracheids (blue). The orange arrowheads (a) point to debonding of adjacent cells. Raman images show integration at the lignin‐specific 1600 cm^−1^ wavenumber, and false‐colour images for ToF‐SIMS show the distribution of ions corresponding to lignin and structural polysaccharides. Bars, 10 μm.

Perhaps most striking from the Raman analysis was the absence of all lignin‐related signals in the cell corners and middle lamellae (Figs 3a,b, S4b). Debonding of cell walls was also frequently observed while sectioning leatherwood xylem (Fig. 4a). To analyse this further, we examined the ultraviolet autofluorescence of lignin across adjacent cell walls and compound middle lamellae of neighbouring cells in leatherwood xylem and observed a drop in the lignin content within the middle lamella between vessels, fibres, and, somewhat less consistently, between vasicentric tracheids (Fig. 4b). By contrast, lignin autofluorescence peaked within the middle lamella between vessels and fibres in aspen (Fig. 4e,f) and between tracheids in spruce (Fig. 4i,j).

Similarly, a weaker response of the lignin‐specific 1600 cm^−1^ Raman signal was observed in middle lamellae between leatherwood fibres, vessels, and tracheids (Fig. 4c). As expected, aspen and spruce again showed the strongest lignin signal in the middle lamella (Fig. 4g,k).

ToF‐SIMS imaging was also used to confirm that leatherwood possesses lignin‐deficient middle lamellae. Although all three species showed characteristic signals for structural polysaccharides in the cell wall, only aspen and spruce exhibited lignin‐related peaks in the middle lamella and cell corners (Fig. 4d,h,l).

Finally, examination of leatherwood samples collected from across the range of *D. palustris* as well as the three congener leatherwood species demonstrated that lignin‐deficient middle lamellae is a trait that is conserved throughout the genus *Dirca* (Fig. S5; Notes S1).

## Discussion

Although lignin biogenesis has been studied extensively and great strides have been made, gaps remain in our understanding of this complex biological process. Fundamental questions persist, including: What is the role of lignin in the middle lamella? Is there a functional significance of lignin structural diversity? What are the evolutionary origins of lignification? And, how is lignification initiated in the first place? To address these and other ongoing topics in lignin biology, new models are sorely needed (Neutelings, 2011).

### Unusual cell wall composition and lignin distribution

In this study, we have shown that leatherwood, a slow‐growing woody shrub, contains conspicuously low levels of lignin and displays a unique distribution of lignin throughout the xylem and across the cell wall. Compared with other woody angiosperms reported in the literature, leatherwood is among the lowest in terms of lignin content (Pettersen, 1984). This is consistent with previous reports that senescent leatherwood leaves contain less lignin than all other shrubs and trees examined (Ferrari, 1999; Jo *et al.*, 2016).

Leatherwood is a member of the family Thymelaeaceae in the order Malvales, a diverse group of trees, shrubs, and lianas, but there is no evidence that poor lignification is a common feature of this family. For example, the Klason lignin contents of *Aquilaria*, *Gonystylus*, and *Daphne* spp. range from 23% to 31% (Syafii & Yoshimoto, 1991; Yamamoto & Hong, 1994; Shrestha & Budhathoki, 2012). Nevertheless, there are family resemblances in histology, including the presence of axial parenchyma and vasicentric tracheids in the xylem and thick bast fibres in the bark (Herber, 2003; Schweingruber *et al.*, 2011; Fig. S6).

Leatherwood xylem contains strands of highly lignified thick‐walled vessels and vasicentric tracheids set amongst thin‐walled fibres that are only weakly lignified (Choquette, 1925). Using histochemical staining and Raman imaging, we found that leatherwood primarily lignifies only the water‐conducting vessels and tracheids, and these cell walls also contain higher levels of guaiacyl lignin and more lignin‐associated aldehyde groups compared to fibres. Such variability in lignin composition may be tailored to differences in cell function, as vessels are specialized for water conduction whereas fibres primarily confer mechanical support (Donaldson, 2001; Pereira *et al.*, 2018). If so, leatherwood xylem may represent an extreme example of cell‐type‐specific lignin specialization, and further investigations could help elucidate the functional significance of lignin structural diversity.

We also found that leatherwood produces a syringyl‐rich lignin and greater levels of cell‐wall‐bound acetyl, two important targets for the improvement of biomass feedstocks in the pulping and biofuel industries. Syringyl lignin content is positively correlated to wood pulpability and biomass pretreatability (Huntley *et al.*, 2003; Mansfield *et al.*, 2012), whereas acetyl levels affect carbohydrate recovery in pretreatment (Johnson *et al.*, 2017). Moreover, acetyl decorations impact xylan deposition and can influence interactions between lignin and other cell wall constituents (Grantham *et al.*, 2017). In this way, acetylation in leatherwood could help provide structural compensation for the lower overall levels of lignin. Raman imaging showed that vasicentric tracheids contain the highest concentration of acetyl groups, perhaps relating to the strengthening role of acetylation in these cell walls (Pawar *et al.*, 2013).

### Wood flexibility and lignin‐deficient middle lamellae

The source of leatherwood's flexibility has remained an open question. Although poor lignification is thought to play a role (Nevling, 1962), others have pointed to the arrangement of vessels (McMinn & Forderhase, 1935, in *D. occidentalis*) and to the shorter fibres, thinner cell walls, and larger cell lumina (Potzger & Geisler, 1937). To this list we can add lower cell wall crystallinity and larger microfibril angles. The largest microfibril angles in leatherwood occur in the walls of vessels (Fig. S4; Notes S1), possibly because these cells must bear the stresses of water conduction.

Although all the aforementioned factors likely contribute to stem flexibility, the occurrence of lignin‐deficient middle lamellae offers the most compelling hypothesis yet. We interrogated the lignin distribution across the compound middle lamella using three analytical techniques and consistently found lignin‐rich middle lamellae in aspen and spruce, but not in leatherwood. Without the rigidity conferred by lignified middle lamellae, it could be that adjacent cells in leatherwood xylem remain free to slip past one another slightly, thereby providing some flexibility to the woody stems.

It has long been known that the cell corners and middle lamellae of mature xylem normally contain the highest concentrations of lignin, accounting for 16–35% of all the lignin in wood (Ritter, 1925; Fergus *et al.*, 1969; Saka & Goring, 1988). And although some heterogeneity in middle lamellar lignin has been reported (Hardell *et al.*, 1980; Singh & Schmitt, 2000; Wi *et al.*, 2005), leatherwood represents the first known example of such a widespread lignin deficiency in the middle lamella.

### Challenging the model of lignification

Reports from diverse plant species have shown that lignification begins in the cell corners and middle lamellae, and then spreads inward to the layers of the cell wall (Donaldson, 2001). Accordingly, it has been hypothesized that the onset of lignin polymerization occurs in these regions (Terashima, 1990; Donaldson, 1994). Putative nucleation sites for lignin initiation include cell‐wall‐bound hydroxycinnamates, pectin, and cell wall proteins (Ralph *et al.*, 1995; Boerjan *et al.*, 2003). Similarly, laccase or peroxidase enzymes that drive lignin polymerization may be specifically tethered to the cell corners and middle lamellae (Boerjan *et al.*, 2003; Chou *et al.*, 2018; Tobimatsu & Schuetz, 2019), but irrefutable evidence for any one mechanism is still lacking. Moreover, previously observed differences in lignin composition between middle lamellae and adjacent cell walls (Whiting & Goring, 1982) are still unexplained.

With cell corners and middle lamellae that remain unlignified in mature xylem, it could be that leatherwood represents an exception to the rule or it may be the case that debunks the model. Accordingly, the lignin distribution should be re‐examined in diverse plant taxa to quell this uncertainty.

Early in the development of xylem tissue, protoxylem cells are formed with bands of lignified secondary cell wall interspersed by regions of primary wall that remain unlignified. This arrangement, which provides axial flexibility for these rapidly growing cells while enabling water conduction, is guided by the deployment of oxidative enzymes specifically to the regions of secondary cell wall (Schuetz *et al.*, 2014). Lateral flexibility in protoxylem is afforded by unlignified middle lamellae, while pectin and cell wall proteins provide cell adhesion (Ryser *et al.*, 2004).

In leatherwood, lignin‐deficient middle lamellae may also confer flexibility, and perhaps this also results from a localized absence of laccase or peroxidase enzymes. Alternatively, the monolignols needed for lignification may not be efficiently delivered to the middle lamella, initiation sites may be lacking in leatherwood, or perhaps radical formation and polymer growth are inhibited by some other means. It is also notable that leatherwood lacks *p*‐hydroxyphenyl moieties, since these lignin units typically predominate in the middle lamella (Whiting & Goring, 1982; Terashima *et al.*, 2012). As the mechanisms undergirding lignin initiation and deposition remain obscure, further studies on the topochemistry of lignification in leatherwood may help elucidate these dynamics and could inform revisions to the classical model.

### Implications of lignin‐deficient middle lamellae

In most plants, lignin‐rich middle lamellae help adjacent xylem cells adhere to prevent slippage. By contrast, leatherwood xylem retains the pectin‐rich middle lamellae and cell corners normally found between unlignified primary walls. While secondary cell wall lignin contributes to compressive strength by increasing the hardness properties of the wall (Niklas, 1992; Gindl *et al.*, 2002), the mechanical role of lignin in the middle lamella is less clear. Compared with secondary walls, lignified middle lamellae have a lower Young's modulus, a similar hardness, and a greater shear strength (Niklas, 1992; Qin *et al.*, 2018), possibly due to the globular macromolecular structures of lignin formed in the absence of cellulose microfibrils (Terashima *et al.*, 2012). Without heavily lignified middle lamellae, leatherwood could be susceptible to xylem fracture. In this light, the clustering of strongly lignified vessels and vasicentric tracheids into oblique strands may be a compensatory adaptation that imparts the requisite shear support with minimal investment in lignin. Similarly, the thick and flexible bark with its strong bast fibres could provide mechanical compensation for a weaker xylem (Holm, 1921). In any case, lignin‐deficient middle lamellae could lead to greater debonding of xylem cells during mechanical shearing (Donaldson, 1995), a highly desirable trait in the processing of woody biomass.

As poor lignification presents a challenge for defence against pest and pathogen, *Dirca* spp. invest in a wide range of defensive compounds, including diterpenes, lignans, coumarins, phenolic glycosides, and a novel class of sulphur‐containing cytotoxins called dirchromones (Badawi *et al.*, 1983; Ramsewak *et al.*, 1999; St‐Gelais *et al.*, 2015). The chemical defence strategy is also used by *Aquilaria* and *Gyrinops* spp., other members of the family Thymelaeaceae whose axial parenchyma produce similar compounds when exposed to wood‐decay microorganisms, resulting in a valuable aromatic xylem called agarwood (Rasool & Mohamed, 2016).

A lignin‐deficiency in the middle lamella could also impact the transport of water and dissolved nutrients through the xylem. Lignin and pectin are highly co‐localized within the middle lamella (Wi *et al.*, 2005; Kim *et al.*, 2015), and this lignin has been negatively correlated to ion‐mediated changes in xylem conductance (Boyce *et al.*, 2004). In this way, lignification of middle lamellae represents a trade‐off between mechanical support and reducing conductance by restricting the accessibility of pectin.

It has been reported that the bordered pits of leatherwood fibres have vestures, while those of vessels and vasicentric tracheids are rarely vestured (Jansen *et al.*, 2000; Dute *et al.*, 2001). As these lignin‐rich protrusions may be involved in regulating water flow and safeguarding against embolisms (Choat *et al.*, 2008), further studies on leatherwood xylem conductance and safety could help elucidate the role of lignin in these important processes.

It remains unclear what selective pressures led leatherwood to develop lignin‐deficient middle lamellae, and it is currently unknown whether this phenomenon occurs in related taxa beyond the genus *Dirca* (Fig. S5). Raman imaging of *Daphne odora* did not uncover similarly unlignified middle lamellae (Zhang *et al.*, 2012), suggesting that leatherwood may be unusual even among the Thymelaeaceae. As lignin represents a substantial investment of photosynthate, this atypical lignification regime undoubtedly imparts considerable energy savings. Although it is not directly useful as a biomass feedstock, leatherwood may soon be enlisted to address ongoing questions in lignin biology and inspire new strategies in lignin engineering.

## Author contributions

YM, ERM and SDM conceived the study. YM, NG and DJ conducted the experiments and analysed the data. YM wrote the manuscript.

## Supporting information


**Fig. S1.** Phylogeny of the family Thymelaeaceae, distribution of *Dirca* spp. in North America, and a photo of showing the flexible woody stems of leatherwood.
**Fig. S2** Principal component analysis of FTIR spectra.
**Fig. S3** Differential interference contrast microscopy images used for microfibril angle measurements.
**Fig. S4** Additional confocal Raman microscopy images of leatherwood.
**Fig. S5** Additional UV microscopy images of *Dirca* spp.
**Fig. S6** Photos of leatherwood.
**Methods S1** Complete methodology for the analysis of lignin and structural polysaccharides.
**Notes S1** Supplemental text describing the FTIR analysis, the microfibril angle measurements, the Raman imaging, and the occurrence of lignin‐deficient middle lamellae across the genus *Dirca*.
**Table S1** Annotations of FTIR bands.
Please note: Wiley Blackwell are not responsible for the content or functionality of any Supporting Information supplied by the authors. Any queries (other than missing material) should be directed to the *New Phytologist* Central Office.
