# Supplementary material for: Atypical lignification in eastern leatherwood (Dirca palustris)
Source: New Phytol. 2020 Feb 3;226(3):704–13. doi: 10.1111/nph.16394 (PMC7187453; doi:10.1111/nph.16394)
Supplement: Supplementary file 1 — Fig. S1. Phylogeny of the family Thymelaeaceae, distribution of Dirca spp. in North America, and a photo of showing the flexible woody stems of leatherwood. Fig. S2 Principal component analysis of FTIR spectra. Fig. S3 Differential interference contrast microscopy images used for microfibril angle measurements. Fig. S4 Additional confocal Raman microscopy images of leatherwood. Fig. S5 Additional UV microscopy images of Dirca spp. Fig. S6 Photos of leatherwood. Methods S1 Complete methodology for the analysis of lignin and structural polysaccharides. Notes S1 Supplemental text describing the FTIR analysis, the microfibril angle measurements, the Raman imaging, and the occurrence of lignin‐deficient middle lamellae across the genus Dirca. Table S1 Annotations of FTIR bands. Please note: Wiley Blackwell are not responsible for the content or functionality of any Supporting Information supplied by the authors. Any queries (other than missing material) should be directed to the New Phytologist Central Office. [file NPH-226-704-s001.pdf]

Supporting Information: Atypical lignification in eastern leatherwood (*Dirca palustris*)

Yaseen Mottiar[1], Notburga Gierlinger[2], Dragica Jeremic[3], Emma R. Master[4], and Shawn D. Mansfield[1]

[1] Department of Wood Science, University of British Columbia, 2424 Main Mall, Vancouver, BC, Canada, V6T 1Z4

[2] Department of Nanobiotechnology, Institute for Biophysics, University of Natural Resources and Life Sciences Vienna, Muthgasse 11, 1190 Wien, Austria

[3] Department of Sustainable Bioproducts, Mississippi State University, Box 9680, Starkville, MS, U.S.A., 39759

[4] Department of Chemical Engineering & Applied Chemistry, University of Toronto, 200 College Street, Toronto, ON, Canada, M5S 3E5

Author for Correspondence: Yaseen Mottiar, ymottiar@mail.ubc.ca, +1-778-872-0440

Accepted: December 11, 2019

SI Notes **2**

SI Methods **4**

Table S1 .... **7**

Fig. S1 .... **8**

Fig. S2 .... **9**

Fig. S3 .... **10**

Fig. S4 .... **11**

Fig. S5 .... **12**

Fig. S6 .... **13**

SI References **14**

SI Notes

The *Dirca* genus

The *Dirca* genus comprises four species of slow-growing sciophilous angiosperms: *D. occidentalis* Gray, which is found only in the San Francisco bay region of California; *D. mexicana* Nesom and Mayfield, which is endemic to a single valley in Mexico's Sierra Madre Oriental Mountains; *D. decipiens* Floden, which is limited to Arkansas and Kansas; and *D. palustris* L., which is widespread but uncommon throughout eastern Canada and the United States (Fig. S1a; Schrader & Graves, 2004; Floden *et al*., 2009). Traditionally, leatherwood was used for naturopathic medicines and for natural cordage. Leatherwood stems are so flexible that one can easily tie a woody twig into a knot without it snapping (Fig. S1b; Anderson, 1933).

FTIR analysis

FTIR was initially used to screen for unique chemical features in leatherwood xylem in comparison to aspen and spruce. Since FTIR spectra of powdered wood are notoriously complex due to overlapping peaks from lignin and structural polysaccharides, spectra of chlorite holocellulose and acid-insoluble lignin were also collected (Fig. S2a–c). Principal component analysis (PCA) of the ensuing datasets was used to identify noteworthy chemical features in leatherwood (Fig. S2d–f).

The first principal component of the PCA model generated for powdered wood primarily differentiated aspen from spruce and accounted for over 78 % of the total variance (Fig. S2g). Compared to spruce, aspen was rich in the regions around 1735 and 1240 cm^{-1}. On the other hand, the spruce spectra were more prominent in the signals at 1510, 1385, 1090 and 1020 cm^{-1}. The second principal component captured 21 % of the cumulative variance while distinguishing spruce and aspen from leatherwood (Fig. S2j). Spruce and aspen contained more of the bonds characterised by 1725, 1510, 1270, 1165, 1120, 1060 and 1030 cm^{-1} whereas leatherwood featured comparatively more of the signals at 1760, 1625 and 975 cm^{-1}. Of particular interest is the deficiency in the 1510 cm^{-1} signal corresponding to generic aromatic skeletal vibrations in lignin (Faix, 1991) which points to lower levels of lignin in leatherwood compared to the spruce and aspen samples.

To fully realise the potential of FTIR, it was prudent to consider lignin and holocellulose separately. The first principal component of the PCA model generated for holocellulose captured 94 % of the total variance and primarily distinguished leatherwood from spruce (Fig. S2h). Leatherwood contained more of the bonds associated with 1740, 1245 and a wide region near 1045 cm^{-1} whereas spruce again featured more of the signal at 1510 cm^{-1}. The second principal component, which captured less than 6 % of the total variance, separated aspen from leatherwood and spruce (Fig. S2k). Aspen holocellulose was rich in the bonds associated with 1335, 1165, 1110 and 980 cm^{-1} while leatherwood and spruce produced stronger signals at 1760, 1610, 1510 and 1270 cm^{-1}. Since several of these signals are lignin-specific bands (eg: 1510, 1270 cm^{-1}), small amounts of undigested lignin likely remained in the holocellulose preparations as is often the case with acid chlorite treatment (Browning, 1967). Even still, the over-representation of the signals at 1740 and 1245 cm^{-1} in leatherwood point to an unusual hemicellulose composition. This was explored further by curve fitting (Fig. 1a,c).

The first component of the PCA model generated for acid-insoluble lignin separated aspen from spruce and leatherwood, and accounted for about 65 % of the total variance (Fig. S2i). It revealed that lignin from aspen contained more of the signal at 1165 and a wide band at 1110 cm^{-1} whereas lignin from spruce and leatherwood contained more of peaks at 1510 and 1270 cm^{-1}. The second principal component captured about 34 % of the total variance and primarily distinguished spruce from leatherwood (Fig. S2l). Lignin from spruce was rich in the peaks at 1510, 1270, 1140 and 1030 cm^{-1} whereas leatherwood contained more of the bonds related to 1720, 1670, 1545 and 1110 cm^{-1}. The weaker signal at 1510 cm^{-1} again points to lower lignin levels in leatherwood compared to aspen and spruce. In addition, the lower abundance of the band near 1270 cm^{-1} points to a noteworthy lignin composition in leatherwood since this

wavenumber is associated with guaiacyl-type lignin (Faix, 1991). This was explored further by curve fitting (Fig. 1b,d).

Microfibril angle

Differential interference contrast microscopy was used to measure the microfibril angle of xylem cells following salt impregnation and sonication (Fig. S3). For each species, 100 measurements were taken for fibres (leatherwood and aspen) and tracheids (spruce). The microfibril angle in leatherwood xylem was consistently higher than aspen and spruce, suggesting that leatherwood cell walls could have greater tensile strength. This comes despite the leatherwood xylem samples being older (it is a slow-growing species) than aspen or spruce. In addition, leatherwood xylem cells are short compared to other woody species (Potzger & Geisler, 1937). The extent to which high microfibril angles in leatherwood xylem contribute to stem flexibility warrants future investigation.

Additional Raman imaging

Closer examination of additional Raman bands can be informative. For example, the signal at 2920 cm^{-1} corresponds to the C–H stretching mode of organic compounds. Integrating this band shows a slightly higher density of structural components in the cell walls of vessels and vasicentric tracheids compared to fibres (Fig. S5a). Poor signals in the middle lamella and cell corners are also apparent. The syringyl lignin-sensitive signal at 1335 cm^{-1} was apparently evenly distributed (Fig. S5b). Although this could indicate a uniform syringyl lignin distribution, interpretation of this signal must be done cautiously due to interference from the nearby cellulose-specific band at 1380 cm^{-1}. The total lignin and guaiacyl lignin signals are much more informative (Fig. 3a,b). The even signal at 380 cm^{-1} shows that similar levels of cellulose were present in vessels, tracheids and fibres (Fig. S5c). Finally, the band at 1090 cm^{-1} can be used to probe cellulose microfibril organisation (Gierlinger *et al*., 2010) and the stronger response in vessels points to higher microfibril angles (Fig. S5d).

Lignin distribution in other *Dirca* species

To ascertain whether lignin-deficient middle lamellae is a trait that is common throughout the genus *Dirca*, additional leatherwood stem samples were examined by confocal microscopy. Firstly, samples of *D. palustris* collected from Maine, Massachusetts and North Dakota were sectioned and examined. All three showed a deficiency of lignin in the middle lamella (Fig. S6a–c). Next, samples of *D. occidentalis* and *D. decipiens* were collected from the natural ranges in California and Kansas. Since the natural range of *D. mexicana* in Mexico is difficult to access, samples were obtained from cultivated specimens. Samples of all three species were sectioned and examined, and all exhibited lignin-deficient middle lamellae (Fig. S6d–f) suggesting that this trait is conserved across the genus *Dirca*.

SI Methods

Sample processing

Xylem samples used for the analysis of lignin, cellulose, acetyl and structural polysaccharides were debarked, air-dried and ground in a Wiley mill to pass a 40-mesh sieve (420 μm). Similarly, samples for FTIR and X-ray diffraction were comminuted to pass a 270-mesh sieve (53 μm).

Fourier-transform infrared spectroscopy

Samples of wood flour, acid-insoluble lignin and acid chlorite-derived holocellulose (2 mg) were homogenised with FTIR-grade potassium bromide (200 mg) in a mortar and pestle and dried overnight at 105 ºC prior to being formed into compressed discs using a hydraulic press and a die mold. Chlorite holocellulose was prepared for each species according to the procedure described by Browning (1967) using sodium chlorite and acetic acid, and acid-insoluble lignin was retained from the lignin quantification procedure described below. Spectra were recorded using a Tensor 27 FTIR spectrometer (Bruker Optics, Ettlingen, Germany) at a resolution of 4 cm^{-1} with 150 scans from 4000 to 400 cm^{-1}. The spectra were corrected for atmospheric contributions, cropped to the fingerprint region (1800 to 800 cm^{-1}) and baseline-adjusted with the rubberband algorithm and 64 baseline points using the OPUS version 5.1 software package (Bruker Optics). Triplicate samples were processed for each species and each sample type.

PCA analysis was performed for all three sample types using the PLS Toolbox Version 5.8 for Matlab (Eigenvector Research Incorporated, Wenatchee, WA, U.S.A.). For each of the three sample types, three spectra from each species were normalised by unit area prior to analysis. For the lignin and holocellulose spectra, peaks were identified using the second derivative scanning mode of OPUS version 5.1 with 9 smoothing points. Curve fitting was then performed using the Levenberg-Maquardt algorithm with variable peak intensity and width, with the peak positions fixed and with the peaks modelled by a pseudo-Voigt shape (50 % Gaussian, 50 % Lorentzian).

Lignin content

The lignin content was quantified using a standardised acid hydrolysis procedure for Klason lignin (Coleman *et al.*, 2008). Briefly, 40-mesh wood powder was Soxhlet-extracted using acetone for 24 hours. Extractive-free wood powder (200 mg) was oven-dried at 105 ºC and weighed prior to treatment with 72 % sulphuric acid (3 mL) for two hours at 20 ºC with stirring every 10 minutes followed by acid hydrolysis in 4 % sulphuric acid (dilution with 112 mL deionied water) for one hour in an autoclave at 121 ºC and 101 kPa. Acid-insoluble lignin was determined gravimetrically as the weight of residue remaining upon medium coarseness sintered-glass crucibles after filtering and drying in an oven at 105 ºC. Acid-soluble lignin was estimated by UV-spectroscopy of the acid hydrolysis filtrates using an extinction coefficient of 110 L g^{-1} cm^{-1} at 205 nm (Swan, 1965). The oven-dried acid-insoluble powder was subsequently used for FTIR analysis, while the acid hydrolysate was used to quantify the structural polysaccharides. Acid-insoluble lignin was corrected for the ash content which was determined as the weight of extractive-free wood powder remaining in porcelain crucibles following four hours at 575 ºC in a muffle furnace. Three biological replicates were analysed for each species using technical triplicates.

Structural polysaccharides composition

The composition of structural polysaccharides was assessed by UHPLC-DAD of acid hydrolysates following reductive amination with benzocaine as described previously (Sakamoto et al., 2015), but with slight modifications. Derivatising reagent (80 μL) containing 100 mg mL^{-1} benzocaine, 117 μL mL^{-1} glacial acetic acid and 10 mg mL^{-1} sodium cyanoborohydride in methanol was added to an aliquot of acid hydrolysate (20 μL) with 3-*O*-methylglucose as an internal standard (5 μL, 1 mg mL^{-1}). The reaction was incubated at 60 °C for 60 minutes and then cooled prior to analysis. Good separation of six neutral sugars and three uronic acids was obtained using an AQUITY BEH C18 column (100 mm × 2.1 mm, 1.7 μm

particle size, Waters Inc., Milford, Massachusetts, U.S.A.) maintained at 50 °C and an Agilent 1290 Infinity II UHPLC apparatus equipped with a diode array detector (Agilent Technologies Inc., Santa Clara, California, U.S.A.). An injection volume of 1 µL was used in non-isocratic separation at 0.5 mL min^{-1} with a gradient of 5 to 12 % acetonitrile in 200 mM borate buffer (pH 8.9) over 10 minutes. Benzocaine-derivatised sugars and uronic acids were detected at the UV maximum of 308 nm, and a column clean-up was performed between samples using 90% acetonitrile in 200 mM borate buffer (pH 8.9). Calibration standards of known concentration enabled quantification and correction for mass loss upon hydrolysis. Three biological replicates were analysed for each species using technical triplicates.

<u>Acetyl content</u>

The cell wall-bound acetyl content was evaluated using HPLC-RI of ester-linked organic acids liberated by alkaline hydrolysis. Extractive-free 40-mesh wood powder (25 mg) was weighed into 2-mL screw-cap vials and 2 M sodium hydroxide (1 mL) was added prior to incubation for 24 hours at 30 ºC and 500 rpm in a ThermoMixer incubator (Eppendorf, Hamburg, Germany). Following incubation, sulphuric acid (100 µL, 72 % w/w) was added and the tubes were mixed by vortexing and placed on ice to terminate the saponification reactions. Finally, acetic acid was quantified by HPLC using a Shodex RI-101 detector and an Aminex HPX-87H column (BioRad Laboratories, Hercules, California) maintained at 60 ºC with a Summit HPLC apparatus (Dionex Corporation, Sunnyvale, California, U.S.A.). An injection volume of 25 µL was employed and good separation of peaks was achieved with an isocratic eluent of 5 mM sulphuric acid at a flow rate of 0.6 mL min^{-1}. Three biological replicates were analysed for each species using technical triplicates, and butyric acid was used as an internal standard.

<u>Lignin composition</u>

The lignin composition was measured by thioacidolysis using the standard methodology coupled with gas chromatography (Robinson & Mansfield, 2009). Thioacidolysis was performed using 10-mg samples of 40-mesh extractive-free wood powder in reactions with 1 mL of 2.5 % boron trifluoride etherate and 10 % ethanethiol in freshly distilled dioxane for four hours at 100 ºC with periodic mixing. After stopping the reactions at -20 ºC and adding 400 mM sodium bicarbonate (0.3 mL) to adjust the pH, the reaction products were extracted using dichloromethane (1 mL) and deionised water (2 mL). The organic phase was dried by passing through a loosely packed column of anhydrous sodium sulphate and then evaporated to dryness using a vacuum centrifuge. Samples were resuspended in dichloromethane (0.7 mL) and lignin monomers were derivatised by adding pyridine (20 µL) and N,O-bis(trimethylsilyl) acetamide (100 µL) to sample aliquots (20 µL). Finally, derivatised monomers were analysed using a Trace 1310 gas chromatography apparatus (Thermo Scientific, Waltham, Massachusetts, U.S.A.) equipped with a TraceGOLD 5MS column using helium as a carrier gas at a flow rate of 1 mL min^{-1}, and a flame ionisation detector fed by hydrogen (35 mL min^{-1}) and compressed air (350 mL min^{-1}). A 1-µL aliquot was injected by splitless injection at 250 ºC and the oven conditions were as follows: hold at 130 ºC for 3 min, then ramp at 6.5 ºC min^{-1} to 230 ºC and hold for 6 min, and finally ramp at 2.5 ºC min^{-1} to 250 ºC and hold for 10 min. Three biological replicates were analysed for each species using technical triplicates, and an internal standard of tetracosane was added prior to phase separation. Peaks corresponding to derivatised products of *p*-hydroxyphenyl, syringyl and guaiacyl monomers were integrated using the Chromeleon 7 software package (Thermo).

<u>Cellulose content</u>

The cellulose content was determined using the standard methodology for alpha cellulose following delignification (Browning, 1967). Extractive-free wood powder (100 mg) was reacted at 50 ºC for 16 hours with 20 % sodium chlorite (1.5 mL) in sodium acetate buffer (3.5 mL containing 60 mL L^{-1} glacial acetic acid and 1.3 g L^{-1} sodium hydroxide). This was repeated for another 16 hours using fresh reaction mixture. Delignified wood powder was then recovered on a medium coarseness sintered-glass crucible after washing with 1 % acetic acid (100 mL) and acetone (10 mL). After drying at 50 ºC, a portion of the recovered holocellulose (30 mg) was incubated at room temperature for 30 minutes with 17.5 % sodium

hydroxide (2.5 mL), and then diluted to 8.75 % by the addition of deionised water (2.5 mL) and incubated for another 30 minutes. After filtering through a medium coarseness sintered-glass crucible and rinsing with deionized water (100 mL), the powder was soaked in 1 M acetic acid for 5 minutes and then rinsed again with water (100 mL). The alpha cellulose content was then determined gravimetrically after drying at 105 ºC.

Crystallinity

X-ray diffraction of wood powder was performed in reflection mode with a Philips powder X-ray diffractometer comprised of a PW1830 HT generator, a PW1050 goniometer and a PW3710 control unit alongside X'Pert Quantify data capture software (PANalytical B.V., Almelo, The Netherlands). The CuKα radiation generated at 40 kV and 40 mA was primarily monochromatic upon passing through a 15-μm PW1385/00 nickel filter. Scans were obtained from 5 to 55 degrees 2θ with steps of 0.02 degrees every 2.5 seconds. An amorphous profile was evaluated using stable amorphous cellulose prepared by dissolving Avicel PH-101 (50 μm particle size, Sigma-Aldrich, St. Louis, Missouri, U.S.A.) in sulphur dioxide-diethylamine-dimethylsulphoxide and regenerating in water before freeze-drying (Isogai & Atalla, 1991). Triplicate samples were processed for each species. The data were baseline-corrected and the amorphous cellulose curve was scaled to reach each experimental spectrum at only one point (Thygesen *et al*., 2005). The crystallinity index was then evaluated as the fraction of total area above the amorphous profile which is attributable to crystallinity.

Microfibril angle

The microfibril angle was evaluated using differential interference contrast microscopy (Wang *et al*., 2001). Radial longitudinal xylem sections with a thickness of 15 μm were obtained using a sliding block microtome. Sections were incubated in a 5 % (w/v) cobalt chloride solution at 80 °C for 2 hours and then transferred to a 40 kHz sonicating water bath with the same solution for an additional 2 hours. Sections were then rinsed with water, mounted onto glass slides, allowed to dry overnight, and visualised using an Axioskop 2 microscope (Carl Zeiss AG, Oberkochen, Germany) equipped with a DFC 450 C camera (Leica Microsystems GmbH, Wetzlar, Germany). The angle of fractured lamellae of cellulose microfibril bundles was measured relative to the long axis of xylem fibres (leatherwood and aspen) and tracheids (spruce) using ImageJ (National Institutes of Health, Bethesda, U.S.A.), and 100 measurements were averaged for each species.

Table S1. Annotations of FTIR bands.

Wavenumbers (cm^{-1})	Band Assignments	Origin	Notes	References
1760 – 1725	C=O stretching of acetyl or carboxyl groups	Holocellulose	Related to the content of acetyl and uronic acids	1, 2
1720 – 1710	C=O stretching of unconjugated ketone and carboxyl groups	Lignin		3
1675 – 1660	C=O stretching of para-substituted aryl ketone groups	Lignin		3
1640 – 1625	C=O stretching of conjugated groups and absorbed O–H	Holocellulose		4, 5
1605 – 1595	Aromatic skeletal vibrations and C=O stretching	Lignin		3, 6
1560 – 1530	Possibly N-H stretching from small amounts of contaminating proteins	Protein		9
1515 – 1505	Aromatic skeletal vibrations	Lignin	Related to the total lignin content	3, 6
1470 – 1425	Aromatic skeletal vibrations and asymmetrical C–H deformations	Lignin		3
1430 – 1420	Symmetrical CH_2 deformations	Holocellulose	Related to the cellulose crystallinity	7, 8
1385 – 1375	Symmetrical C–H deformations	Holocellulose and Lignin		3, 4, 5
1335 – 1325	Syringyl ring breathing and C–O stretching	Lignin	Related to the guaiacyl lignin content	3
1335 – 1315	In-plane O–H deformations and CH_2 wagging	Holocellulose		4,5
1275 – 1265	Guaiacyl ring breathing and C–O stretching	Lignin	Related to the syringyl lignin content	3
1250 – 1230	C–O stretching of acetyl groups	Holocellulose		1, 2
1170 – 1160	C–O stretching of conjugated ester groups	Lignin		6
1165 – 1125	Asymmetrical vibrations of C–O–C groups	Holocellulose		4, 10
1145 – 1135	Guaiacyl in-plane C–H deformations	Lignin		3
1130 – 1100	Syringyl in-plane C–H deformations	Lignin		3, 5
1115 – 1105	Asymmetrical ring stretching	Holocellulose		4
1090 – 1080	C–O deformation of secondary alcohols and aliphatic ethers	Lignin		3
1060 – 1015	C–O stretching related to cellulose and hemicellulose	Holocellulose		4, 5, 10
1035 – 1030	Guaiacyl in-plane C–H deformations and C–O deformations of primary alcohols	Lignin		3
985 – 970	Out-of-plane trans =CH deformations	Lignin		3
915 – 815	Aromatic C–H out-of-plane deformations	Lignin		3

[1] Marchessault (1962)
[2] Stefke *et al*. (2008)
[3] Hergert (1971)
[4] Fengel & Ludwig (1991)
[5] Pandey (1999)
[6] Faix (1991)
[7] O'Connor *et al*. (1958)
[8] Ciolacu *et al*. (2011)
[9] Stuart (2004)
[10] Kacurakova *et al*. (2000)

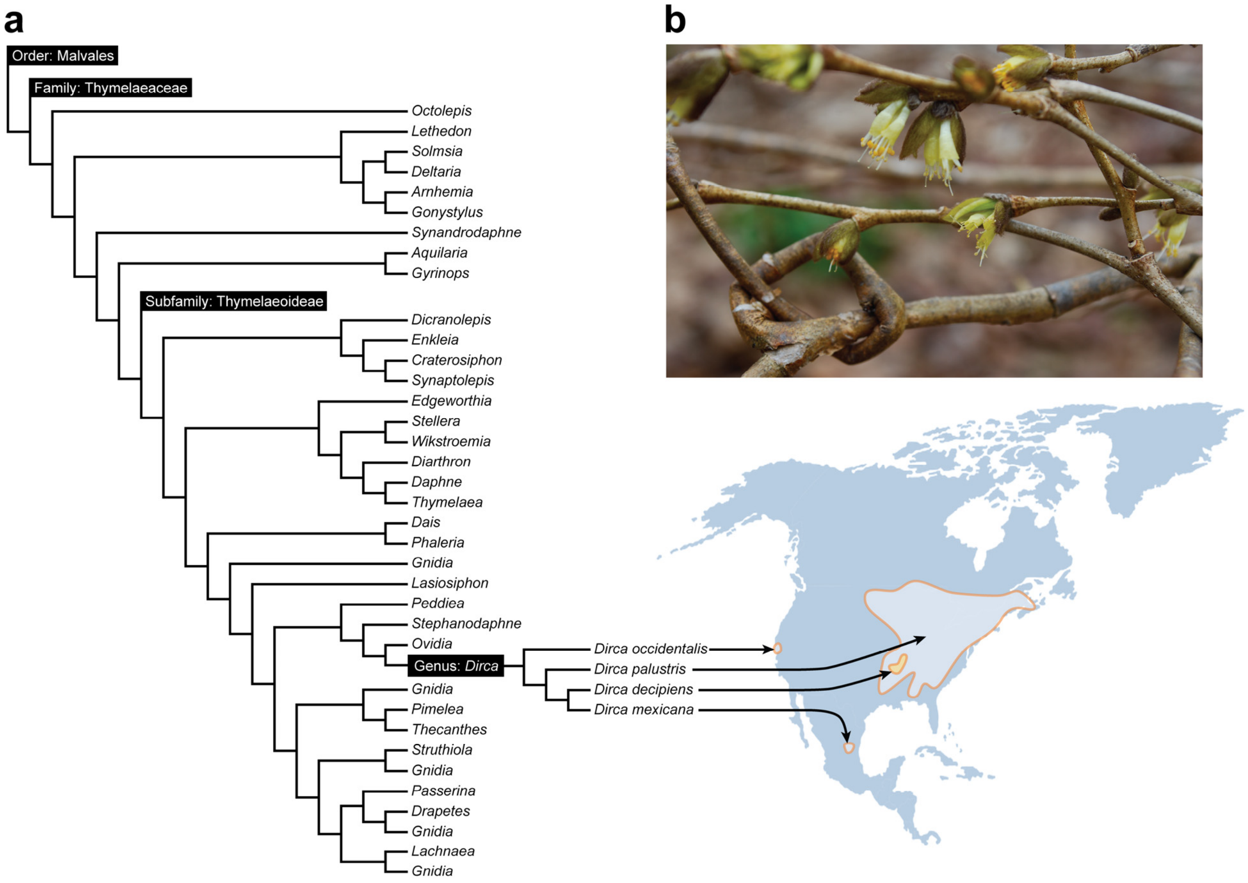

Fig. S1 A partial phylogeny showing eastern leatherwood (*Dirca palustris* L.) within the family Thymelaeaceae and the distribution of the genus *Dirca* in North America *sensu* Floden *et al*., 2009 and Beaumont *et al*., 2009 (a, note that *Gnidia* remains polyphyletic as shown). A flowering branch of leatherwood that was tied in a knot but continues to grow unabated (b).

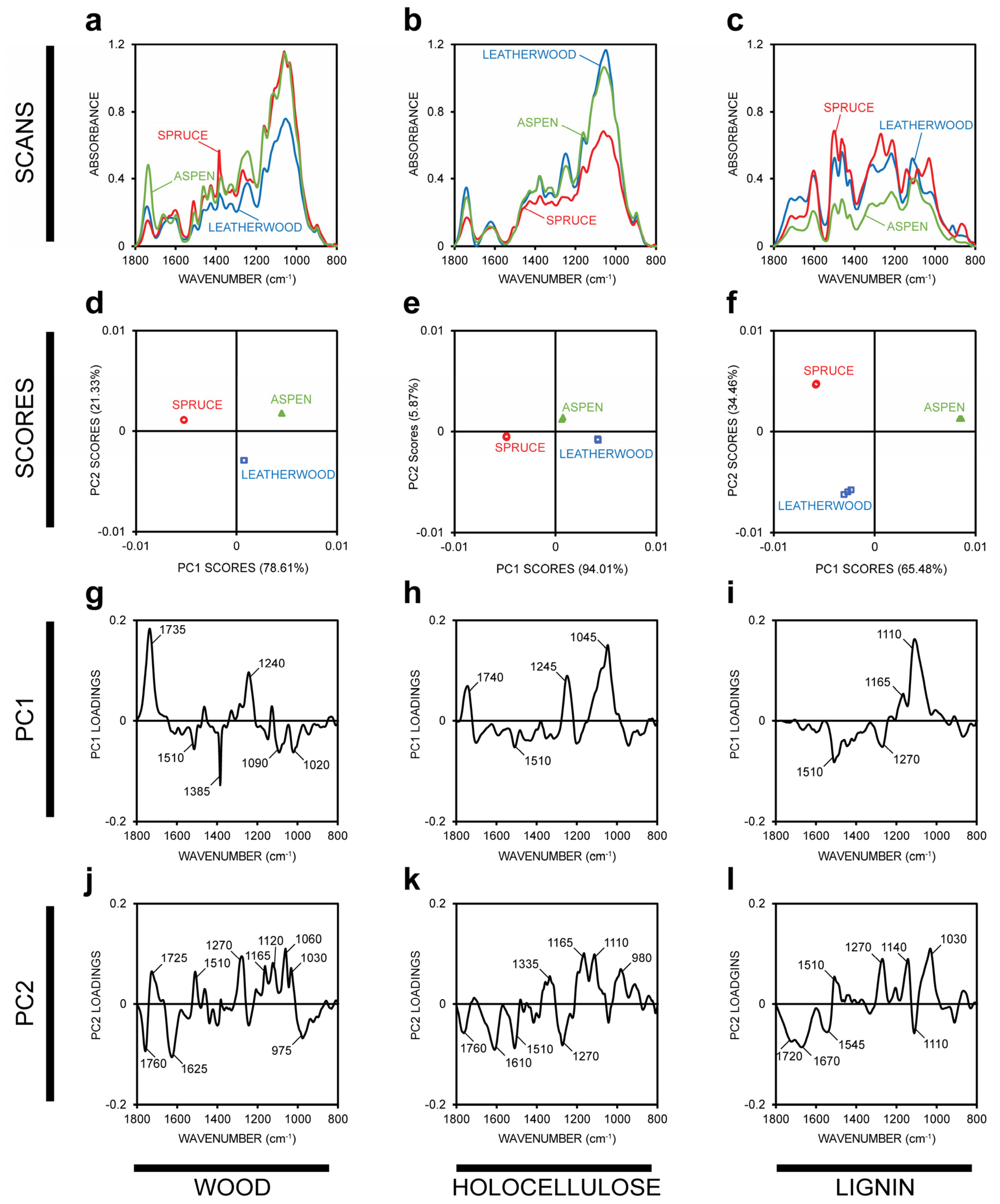

Fig. S2 Principal component analysis for FTIR of powdered wood, chlorite holocellulose, and acid-insoluble lignin. Representative spectra (a–c) are shown for leatherwood (blue), aspen (green) and spruce (red). Models (d–f) captured over 99 % of the cumulative variance in two principal components and are based on triplicate scans for each species and sample type. Loadings corresponding to the first component of the models (PC1) are shown in panels g–i while loadings for the second component (PC2) are shown in panels j–l. Loadings values exceeding ± 0.05 have been labelled with the corresponding wavenumbers.

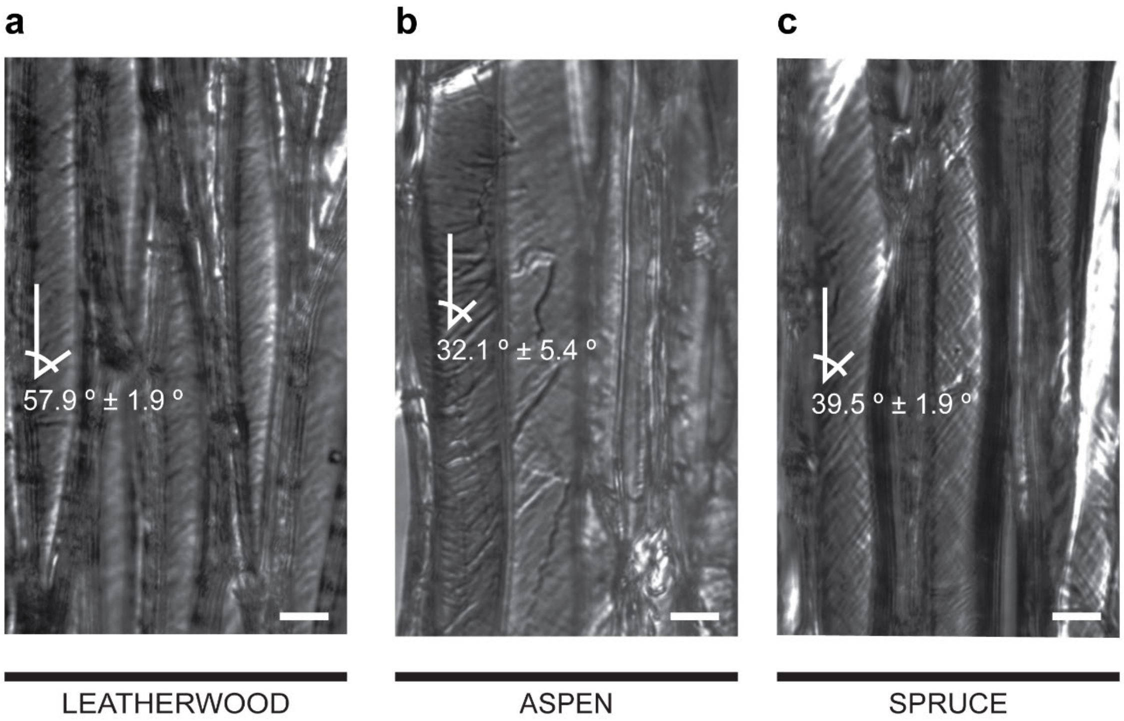

Fig. S3 Longitudinal xylem cross-sections of leatherwood (a), aspen (b) and spruce (c) imaged by differential interference contrast microscopy showing how microfibril angles can be measured from fractured lamellae of cellulose microfibril bundles which become visible after salt impregnation and sonication. The average angle for 100 measurements is shown alongside standard deviation. Scale bars represent 10 μm.

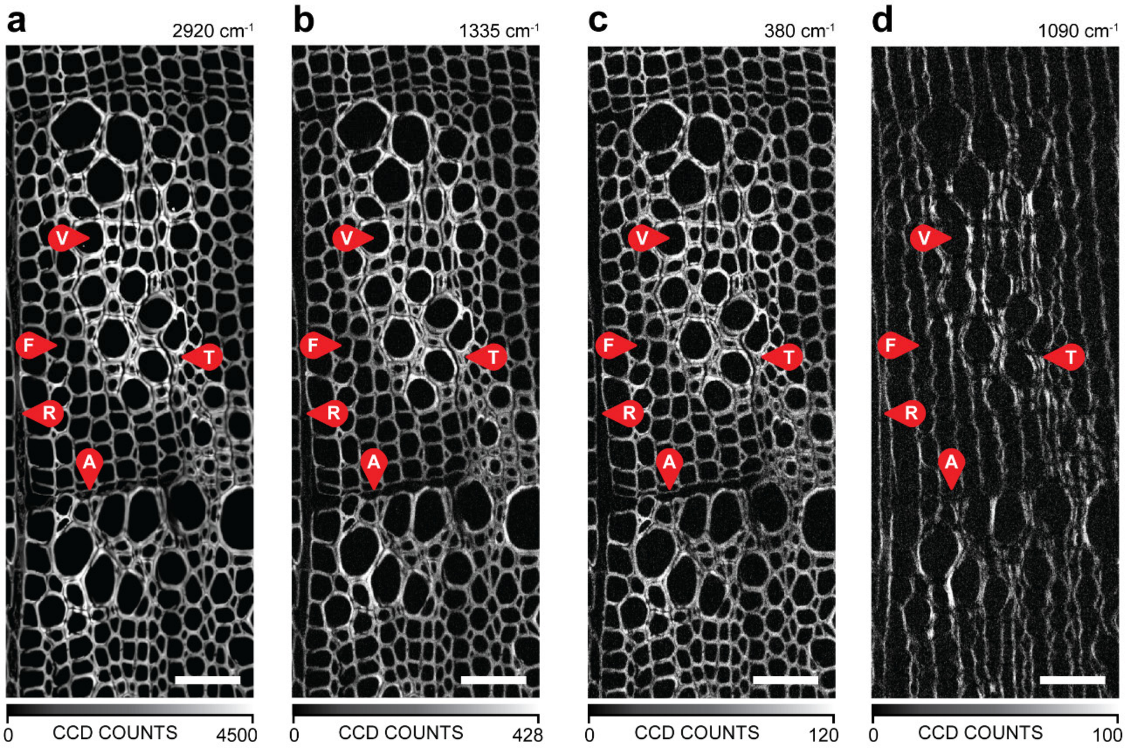

Fig. S4 Transverse cross-sections of leatherwood xylem imaged by confocal Raman microscopy. Chemical images are shown for specific integrated bands corresponding to total organics (a, 2920 cm^{-1}), syringyl lignin (b, 1335 cm^{-1}), cellulose (c, 380 cm^{-1}) and cellulose orientation (d, 1090 cm^{-1}). Images show vessel elements (V), fibres (F), ray parenchyma (R), axial parenchyma (A), and tracheids (T). Scale bars represent 50 μm.

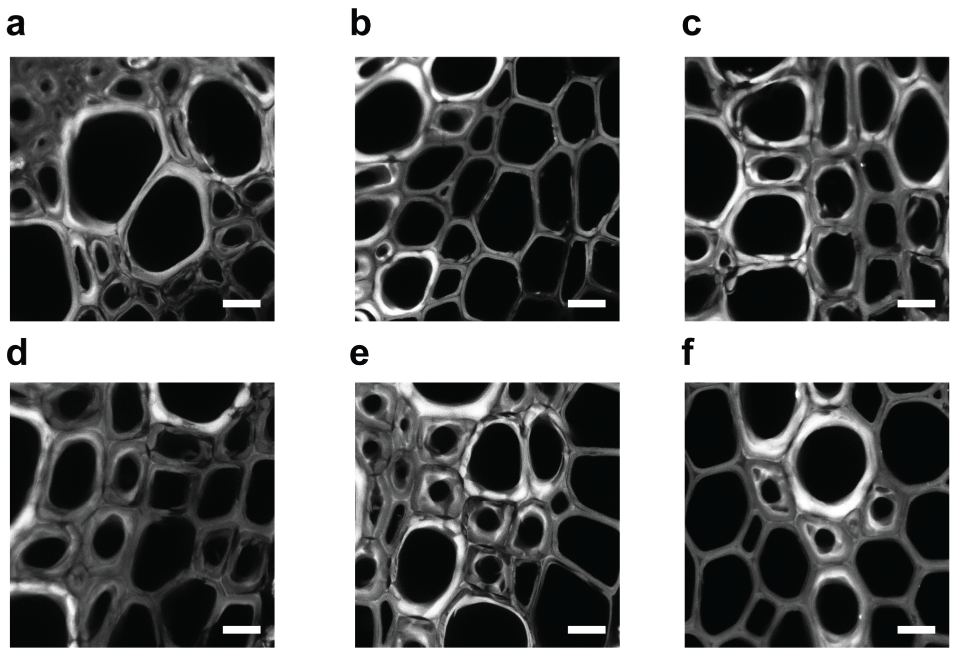

Fig. S5 Lignin-deficient middle lamellae across the genus *Dirca*. Lignin autofluorescence imaged by UV microscopy for transverse xylem cross-sections of *Dirca palustris* from North Dakota (a), Massachusetts (b), and Maine (c), as well as *Dirca occidentalis* from California (d), *Dirca decipiens* from Kansas (e) and *Dirca mexicana* originally from Tamaulipas (f). Scale bars represent 10 μm.

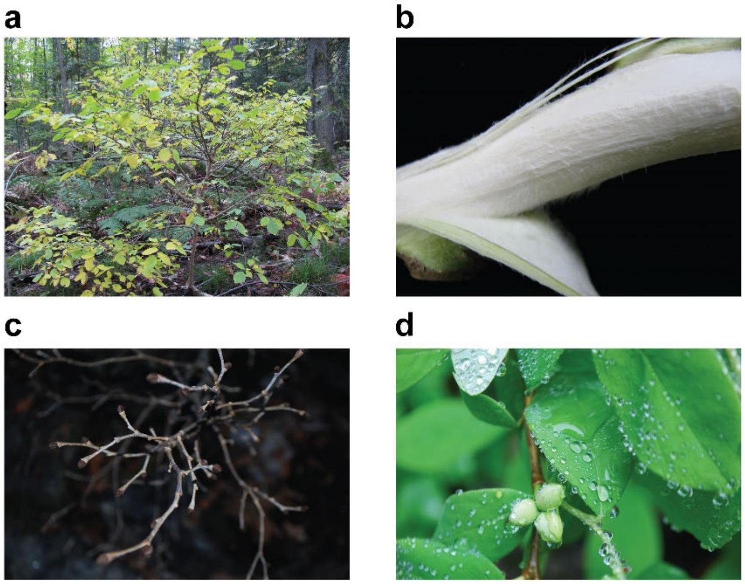

Fig. S6 Photos of leatherwood showing the arboresque growth form (a), the thick bast fibres in the bark (b), the structure of the branches (c), and the leaves and developing drupes (d).

SI References

Anderson E. 1933. Leatherwood (*Dirca palustris*). *Bulletin of Popular Information, Arnold Arboretum, Harvard University* **4**(1, 5): 25–27.

Beaumont AJ, Edwards TJ, Manning J, Maurin O, Rautenbach M, Motsi MC, Fay MF, Chase MW, Van der Bank M. 2009. *Gnidia* (Thymelaeaceae) is not monophyletic: Taxonomic implications for Thymelaeoideae and a partial new generic taxonomy for *Gnidia*. *Botanical Journal of the Linnean Society* **160**: 402–417.

Browning BL. 1967. *Methods of Wood Chemistry*. Volume II. New York, U.S.A.: Interscience Publishers.

Ciolacu D, Ciolacu F, Popa VI. 2011. Amorphous cellulose - Structure and characterization. *Cellulose Chemistry and Technology* **45**: 13–21.

Coleman HD, Park J-Y, Nair R, Chapple C, Mansfield SD. 2008. RNAi-mediated suppression of *p*-coumaroyl-CoA 3'-hydroxylase in hybrid poplar impacts lignin deposition and soluble secondary metabolism. *Proceedings of the National Academy of Sciences of the U.S.A.* **105**: 4501–4506.

Faix O. 1991. Classification of lignins from different botanical origins by FT-IR spectroscopy. *Holzforschung* **45**(S1): 21–27.

Fengel D, Ludwig M. 1991. Möglichkeiten und grenzen der FTIR-spektroskopie bei der charakterisierung von cellulose. Teil 1. vergleich von verschiedenen cellulosefasern und bakterien-cellulose. *Das Papier* **45**: 45–51. [in German]

Floden AJ, Mayfield MH, Ferguson CJ. 2009. A new narrowly endemic species of *Dirca* (Thymelaeaceae) from Kansas and Arkansas, with a phylogenetic overview and taxonomic synopsis of the genus. *Journal of the Botanical Research Institute of Texas* **3**: 485–499.

Gierlinger N, Luss S, König C, Konnerth J, Eder M, Fratzl P. 2010. Cellulose microfibril orientation of *Picea abies* and its variability at the micron-level determined by Raman imaging. *Journal of Experimental Botany* **61**: 587–595.

Hergert HL. 1971. *Infrared spectra*. In: Sarkanen KV, Ludwig CH, eds. Lignins: Occurrence, formation structure and reactions, New York, U.S.A: Wiley-Interscience, 267–297.

Isogai A, Atalla RH. 1991. Amorphous celluloses stable in aqueous media: Regeneration from SO_2-amine solvent systems. *Journal of Polymer Science, Part A, Polymer Chemistry* **29**: 113–119.

Kačuráková M, Capek P, Sasinková V, Wellner N, Ebringerová A. 2000. FT-IR study of plant cell wall model compounds: Pectic polysaccharides and hemicelluloses. *Carbohydrate Polymers* **43**: 195–203.

Marchessault RH. 1962. Application of infra-red spectroscopy to cellulose and wood polysaccharides. *Pure and Applied Chemistry* **5**: 107–129.

O'Connor RT, DuPré EF, Mitcham D. 1958. Applications of infrared absorption spectroscopy to investigations of cotton and modified cottons. Part I: Physical and crystalline modifications and oxidation. *Textile Research Journal* **28**: 382–392.

Pandey KK. 1999. A study of chemical structure of soft and hardwood and wood polymers by FTIR spectroscopy. *Journal of Applied Polymer Science* **71**: 1969–1975.

Potzger JE, Geisler F. 1937. Some physical features associated with pliability of wood. *Proceedings of the Indiana Academy of Science* **46**: 88–92.

Robinson AR, Mansfield SD. 2009. Rapid analysis of poplar lignin monomer composition by a streamlined thioacidolysis procedure and near-infrared reflectance-based prediction modeling. *Plant Journal* **58**: 706–714.

Sakamoto S, Yoshida K, Sugihara S, Mitsuda N. 2015. Development of a new high-throughput method to determine the composition of ten monosaccharides including 4-*O*-methyl glucuronic acid from plant cell walls using ultra-performance liquid chromatography. *Plant Biotechnology* **32**: 55–63.

Schrader JA, Graves WR. 2004. Systematics of *Dirca* (Thymelaeaceae) based on ITS sequences and ISSR polymorphisms. *SIDA, Contributions to Botany* **21**: 511–524.

Stefke B, Windeisen E, Schwanninger M, Hinterstoisser B. 2008. Determination of the weight percentage gain and of the acetyl group content of acetylated wood by means of different infrared spectroscopic methods. *Analytical Chemistry* **80**: 1272–1279.

Stuart BH. 2004. *Infrared spectroscopy: Fundamentals and applications*. Chichester, U.K.: Wiley, 71–93.

Swan B. 1965. Isolation of acid-soluble lignin from the Klason lignin determination. *Svensk Papperstidning* **68**: 791–795.

Thygesen A, Oddershede J, Lilholt H, Thomsen AB, Ståhl K. 2005. On the determination of crystallinity and cellulose content in plant fibres. *Cellulose* **12**: 563–576.

Wang HH, Drummond JG, Reath SM, Hunt K, Watson PA. 2001. An improved fibril angle measurement method for wood fibres. *Wood Science and Technology* **34**: 493–503.
